# Chemical and enantioselective GC–MS characterization of *Minthostachys mollis* (Benth.) Griseb. Essential oil from Peru and its acetylcholinesterase inhibitory activity

**DOI:** 10.3389/fphar.2026.1864128

**Published:** 2026-06-24

**Authors:** Oscar Herrera-Calderon, James Calva, Juan Manuel Guzmán-Flores, Gilmar Peña-Rojas, Vidalina Andia-Ayme, Eddie Loyola-Gonzales, Josefa Bertha Pari-Olarte, José Santiago Almeida-Galindo

**Affiliations:** 1 Department of Pharmacology, Bromatology and Toxicology, Faculty of Pharmacy and Biochemistry, Universidad Nacional Mayor de San Marcos, Lima, Peru; 2 Departamento de Química, Universidad Técnica Particular de Loja (UTPL), Loja, Ecuador; 3 Departamento de Ciencias de la Salud, Centro Universitario de Los Altos, Universidad de Guadalajara, Guadalajara, Jalisco, Mexico; 4 Faculty of Biological Science, Universidad Nacional de San Cristóbal de Huamanga, Ayacucho, Peru; 5 Faculty of Pharmacy and Biochemistry, Universidad Nacional San Luis Gonzaga, Ica, Peru; 6 Faculty of Medicine, Universidad Nacional San Luis Gonzaga, Ica, Peru

**Keywords:** acetylcholinesterase, Alzheimer’s disease, aromatic plants, enantiomers, essential oil, Lamiaceae, molecular docking

## Abstract

**Background:**

Minthostachys mollis (Benth.) Griseb., an aromatic medicinal plant widely used in traditional medicine in Peru, was investigated using an integrated chemical, enantioselective, and acetylcholinesterase (AChE) inhibitory approach.

**Aim:**

To characterize the chemical and enantiomeric composition of M. mollis essential oil by GC–MS, evaluate its AChE inhibitory activity in vitro, and explore the molecular interactions of its volatile constituents with human AChE through molecular docking and molecular dynamics simulations.

**Methods:**

The essential oil (EO) collected from the leaves via steam distillation was analyzed using Gas Chromatography-Mass Spectrometry (GC–MS). Enantioselective GC–MS analysis was performed for the first time on this species. AChE inhibitory activity was evaluated in vitro using Ellman’s assay, and molecular docking and molecular dynamics studies were performed on human AChE (PDB: 4EY7).

**Results:**

GC–MS analysis revealed 33 volatile compounds, of which 30 were structurally identified, accounting for 100% of the total EO composition. Oxygenated monoterpenes were the predominant chemical class (86.24%), with pulegone (63.82%) as the major constituent, followed by carvacrol (5.52%), 1,8-cineole (5.50%), and (E)-isocitral (4.98%). Enantioselective analysis revealed a marked stereochemical preference, including enantiomerically pure (S)-(−)-limonene and (1R,5R)-(+)-sabinene, as well as the predominance of (1S,5S)-(−)-α-pinene (92.54%, e.e. = 84.97%) and (1S,5S)-(−)-β-pinene (80.63%, e.e. = 61.86%). The AChE inhibitory activity showed a moderate inhibitory effect of the EO, with an IC_50_ value of 466.4 ± 1.01 μg/mL. Sesquiterpenes such as γ-muurolene, δ-amorphene, and β-bourbonene exhibited the most favorable binding affinities (ΔG values ranging from −7.3 to −7.0 kcal/mol), mainly mediated by hydrophobic interactions within the catalytic and peripheral anionic sites.

**Conclusion:**

This study provides a comprehensive chemical and stereochemical characterization of M. mollis EO, supporting its relevance as a source of bioactive volatile compounds with moderate AChE inhibitory activity.

## 1 Introduction

Alzheimer’s disease (AD) is a neurodegenerative disease and the leading cause of dementia in individuals aged ≥65 years. AD is characterized by the accumulation of beta-amyloid (Aβ) plaques and tau protein abnormalities (Scarano et al., 2025). Acetylcholinesterase (AChE) plays a role in AD pathology by hydrolyzing acetylcholine, a neurotransmitter essential for memory and learning processes (Li et al., 2024; Grabowska et al., 2025). In patients with AD, cholinergic transmission is decreased, contributing to cognitive impairment. Consequently, AChE inhibitors such as donepezil and galantamine, are widely used in symptomatic treatment to enhance cholinergic signaling and improve cognitive performance (Li, 2022; Zueva et al., 2023). Accordingly, the search for new AChE inhibitors from natural sources has gained increasing interest as a strategy for identifying alternative therapeutic agents for AD treatment.

**FIGURE 1:**
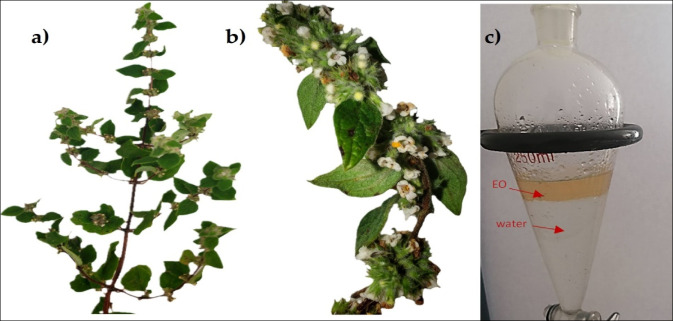
**(a)** The aerial parts of *M. mollis*, **(b)** The inflorescence, **(c)** The EO of *M. mollis*.

*Minthostachys mollis* (Benth.) Griseb. (Lamiaceae), known as “muña” in Peru (Figure 1), is an aromatic plant native to Venezuela, Bolivia, Colombia, Ecuador, and Peru. This climbing shrub predominantly flourishes in subtropical regions. It is used for medicinal purposes and as a natural food additive *(Plants of the World Online)*. In traditional medicine, it is consumed as an infusion to treat digestive ailments, such as flatulence, nausea, colic, and diarrhea. Other uses include sedatives, analgesics for headaches, and treatment of respiratory ailments such as colds and coughs (Schmidt-Lebuhn, 2008). This aromatic plant has been used since ancient times, and in some places in Peru, it has been used as a potato tubers preservative. Furthermore, the essential oils of *M. mollis* have been commercialized as anti-inflammatory and analgesic agents (Otoya, 2020). Some studies have reported that *M. mollis* exhibits antioxidant, antimicrobial (Quispe-Sanchez et al., 2025), and antifungal activities against *Trichophyton tonsurans, Trichophyton mentagrophytes, Microsporum canis* (Cano et al., 2008), and *Candida albicans* (Huamaní et al., 2021).

Although *M. mollis* has not been traditionally used specifically for cognitive disorders, aromatic plants rich in terpenoids have attracted considerable interest owing to their neuroprotective potential, including antioxidant, anti-inflammatory (Kim et al., 2020; Siddiqui et al., 2024), and acetylcholinesterase (AChE) inhibitory activities, which are relevant in the context of neurodegenerative diseases such as AD (Wojtunik-Kulesza et al., 2017). In addition, several essential oils (EOs) from aromatic plants have been studied for their potential effects on AD using *in silico* and *in vitro* models. For instance, the EOs from *Salvia officinalis*, *Salvia lavandulifolia*, *Melissa officinalis*, *Lavandula angustifolia*, and *Rosmarinus officinalis* have demonstrated promising results exhibiting properties anti-amyloid, antioxidant, anticholinesterase, and memory-enhancing (Benny and Thomas, 2019).

Regarding its chemical composition, previous studies have revealed that *M. mollis* EO exhibits high chemical variability depending on the geographical origin and environmental conditions. Two main chemotypes have been described in the literature, one chemotype dominated by pulegone and menthone, and the other characterized by carvacrol and thymol derivatives. Notably, the pulegone–menthone chemotype is the most frequently reported in species from Peru, where pulegone is often the major volatile constituent of the EO (León-Vargas et al., 2026). These variations in the chemical composition may influence its biological properties. In addition, toxicological studies have reported that *M. mollis* EO exhibits a median lethal dose of approximately 500 mg/kg in rats (Rojas-Armas et al., 2019).

Although the chemical composition of *M. mollis* EO has been reported in several geographic regions, its stereochemical composition remains unexplored, and studies focused on its interaction mechanisms with AChE remain limited. In this context, the present study combined enantioselective GC–MS analysis with *in vitro* AChE inhibition assays and computational approaches, to evaluate the neuropharmacological potential of this EO. Therefore, this investigation was designed as an exploratory study integrating chemical, biological, and computational methodologies. The study included the following: (i) macroscopic examination of the plant leaves, (ii) organoleptic evaluation and chemical characterization by GC–MS, (iii) experimental evaluation of AChE inhibitory activity *in vitro*, and (iv) molecular docking against human AChE co-crystallized with donepezil (PDB ID: 4EY7), followed by molecular dynamic simulations of the best-ranked compounds.

## 2 Materials and methods

### 2.1 Collection and extraction of M. mollis EO

Fresh leaves were collected in June 2025 from naturally occurring *M. mollis* plants in the Vinchos district, Province of Huamanga, Department of Ayacucho, Peru (−13.2875, −74.3128; 3700.5 m. a.s.l.). A voucher specimen (119-USM-MHN-2025) was deposited at the Herbarium of the Universidad Nacional Mayor de San Marcos. The EO was obtained by steam distillation using a semi-industrial stainless-steel distillation unit. Approximately 2.5 kg of dried leaves were placed on a mesh above boiling water, allowing the generated steam to pass through the plant material without direct contact with water (Spinozzi et al., 2025). The vapor generated in the apparatus was condensed, and the oil phase was subsequently separated using a separatory funnel and stored at 4 °C until further analysis.

### 2.2 The macroscopic study

Macroscopic examination of the plant material was performed using a trinocular stereomicroscope (SMZ745, Nikon Corporation, Tokyo, Japan), which allowed the observation of morphological characteristics, such as shape, color, surface texture, and the presence of trichomes or glandular structures.

### 2.3 Gas chromatography analysis

Qualitative analysis of the volatile chemical constituents of *M. mollis* EO was performed using a gas chromatography–mass spectrometry (GC-MS) system, the Trace 1310 model from Thermo Fisher Scientific (Waltham, MA, USA), coupled with a single quadrupole mass spectrometer (MS) model ISQ 7000. Separation was achieved using a nonpolar capillary column composed of 5% phenyl-methylpolysiloxane (30 m *×* 0.25 mm, 0.20 μm film thickness) from Thermo Fisher Scientific (Waltham, MA, USA). The samples were injected in split mode (40:1) by adding 1 μL–1000 µL of cyclohexane solution (1:100). The oven program began at 50 °C (held for 3 min), increased at 3 °C/min to 230 °C. The MS detector operated in the full-scan mode (40–350 m/z). The volatile components of the EO were identified by comparing their mass spectra with those of reference compounds that showed similar linear retention indices (LRIs) and those reported in the literature (Mejia-Ramos et al., 2025). The LRIs were calculated and matched against a homologous series of n-alkanes C9-C25 (Sigma–Aldrich, St. Louis, MO, USA). A compound was considered positively identified when the LRI differed by no more than ±20 units from the corresponding values in the literature (Adams, 2017).

Quantitative analysis was performed using the same equipment as that used in the GC-MS, the only difference was that we used a flame ionization detector (GC-FID). The FID was fed with a gas mixture composed of hydrogen (35 mL/min) and air (350 mL/min) and maintained at a final temperature of 230 °C, operating with the same method, and without using a correction factor. All analyses were performed in triplicates.

### 2.4 Enantioselective analysis

Enantioselective analysis of the chiral compounds present in *Minthostachys mollis* EO was performed using gas chromatography coupled with mass spectrometry (GC-MS) and a capillary column with a 2,3-diacetyl-6-tert-butyl-dimethylsilyl-β-cyclodextrin (DAC) stationary phase, which demonstrated the ability to resolve the enantiomeric pairs of interest. The instrumental conditions employed included an injector and transfer line at 220 °C, a constant carrier gas pressure of 70 kPa, and a thermal program of 50 °C (1 min) to 220 °C (10 min) with a ramp of 2 °C/min, which enable the separation and quantification of eight chiral compounds, including α-pinene, sabinene, β-pinene, limonene, and linalool.

The identification of the chemical structures was confirmed by comparing the mass spectra and LRIs with commercially available enantiomerically pure standards, some of which were purchased from Merck (Sigma-Aldrich, St. Louis, MO, USA). The limited availability of other standards restricts the number of compounds that can be analyzed (Mejia-Ramos et al., 2025).

### 2.5 Acetylcholinesterase (AChE) inhibition of M. mollis EO in vitro

AChE inhibition activity was assessed with a spectrophotometric method using the Ellman’s assay. The reaction mixture included 40 *µ*L Tris buffer, 20 *µ*L EO, 20 *µ*L acetylthiocholine, and 100 *µ*L DTNB reagent. Donepezil hydrochloride (Sigma-Aldrich, St. Louis, MO, USA) was used as the positive control. The EOs were pre-incubated at 25 °C for 3 min with constant stirring. Subsequently, 20 *µ*L AChE (0.5 U/mL) was added to initiate the reaction. The reaction was monitored using an EPOCH 2 microplate reader (BIO-TEK, Winooski, VT, USA) at 405 nm for 60 min at 25 °C. Lastly, 10 mg of EO was dissolved in 1 mL of MeOH, and further dilutions were made to achieve final concentrations of 1000, 100, and 10 *μ*g/mL, respectively. The final concentrations of *M. mollis* EO in the reaction wells were 100, 10, and 1 μg/mL, based on a 1:10 dilution factor resulting from the addition of 20 µL of each stock solution (1000, 100, and 10 μg/mL) to a total reaction volume of 200 µL (Calva et al., 2025; Mejia-Ramos et al., 2025).

### 2.6 In silico analysis

The three-dimensional crystal structure of human acetylcholinesterase (AChE; PDB ID: 4EY7) was retrieved from the RCSB Protein Data Bank (https://www.rcsb.org/). Protein preparation was performed using BIOVIA Discovery Studio (Dassault Systèmes, San Diego, CA, USA), which included the removal of crystallographic water molecules, co-crystallized ligands, and other irrelevant heteroatoms. The three-dimensional structures of the essential oil constituents were obtained from the PubChem database (https://pubchem.ncbi.nlm.nih.gov/). Ligand structures were energy-minimized (1000 steps), with added polar hydrogens, and the structures were converted to the PDBQT format for docking calculations. Molecular docking calculations were performed using AutoDock Vina version 1.2.6. The docking protocol was validated by redocking the co-crystallized donepezil into the AChE-binding site. The grid box was centered on the donepezil binding coordinates with dimensions extending 5 Å from the ligands. For each ligand, 20 docking poses were generated, and the mean binding free energy (ΔG) was determined. Docking conformations and protein–ligand interaction profiles were analyzed and visualized using BIOVIA Discovery Studio. Additionally, to evaluate the potential influence of stereochemical configuration on AChE binding, molecular docking was performed independently for both enantiomers of α-pinene, β-pinene, and linalool, as well as for the single enantiomers detected for limonene [(S)-(−)] and sabinene [(1R,5R)-(+)], using the same protocol described above.

### 2.7 Molecular dynamics analysis

Molecular dynamics (MD) simulations were performed using YASARA Structure software (version 25.1.13, YASARA Biosciences GmbH, Vienna, Austria) to evaluate the structural stability of the γ-muurolene–AChE complex, which exhibited the most favorable binding energy in docking analysis. A 100 ns production MD simulation was performed using the md_run.mcr macro (https://www.yasara.org/md_run.mcr). The system was parameterized using the default AMBER14 force field, and the TIP3P explicit water model was employed for the solvation. The complex was placed in a cubic simulation box under periodic boundary conditions with a water density of 0.997 g/mL. Physiological conditions were simulated at pH 7.4, 0.9% NaCl, 310 K, and constant pressure. The trajectories in MD were recorded every 100 ps in. sim format. The structural stability was assessed by calculating the root mean square deviation (RMSD) of the protein backbone atoms and the root mean square fluctuation (RMSF) of individual residues using the md_analyze.mcr macro (https://www.yasara.org/md_analyze.mcr) described by Krieger and Vriend (2015).

### 2.8 Statistical analysis

All experiments were performed in triplicate, and the results were expressed as mean ± standard deviation (SD). The half-maximal inhibitory concentration (IC_50_) values for AChE inhibition assay were calculated from the concentration-response curves using nonlinear regression with a four-parameter logistic model using GraphPad Prism software version 10.1.2 (San Diego, CA, USA).

## 3 Results

### 3.1 Stereomicroscopic study

**FIGURE 2:**
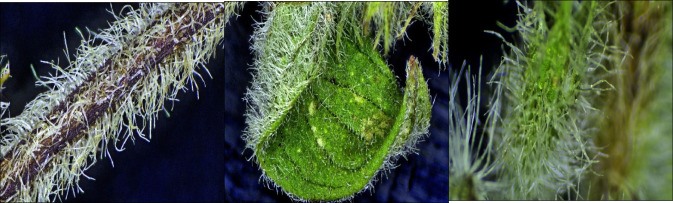
Stereomicroscopic images of *M. mollis* stems and leaves. The stem surface (left) shows a dense covering of long, translucent, uniseriate, and non-glandular trichomes. The young leaf (center) exhibited a similar indumentum, with abundant filamentous trichomes distributed along the margins and on the adaxial and abaxial surface. At higher magnification (right), the trichomes displayed a slender, filiform morphology with smooth walls and pointed apices, forming a dense protective layer over the epidermis.

Stereomicroscopic observations revealed that the stems and young leaves of *M. mollis* exhibited a dense indumentum composed predominantly of elongated, uniseriate, non-glandular trichomes (Figure 2). The stem surface was almost completely covered with translucent trichomes, forming a compact layer on the epidermis. Young leaves exhibited a similar distribution pattern, with abundant filamentous trichomes along the margins and adaxial surfaces. At higher magnifications, these trichomes appeared slender and flexible with smooth cell walls and pointed apices. This dense coverage suggests a protective role against environmental stress and possible involvement in limiting water loss and herbivory. Such trichome structures are characteristic of Lamiaceae species and are commonly associated with the biosynthesis and accumulation of essential oils, supporting the relevance in EO-producing plants.

### 3.2 Chemical characterization and GC-MS analysis

**FIGURE 3:**
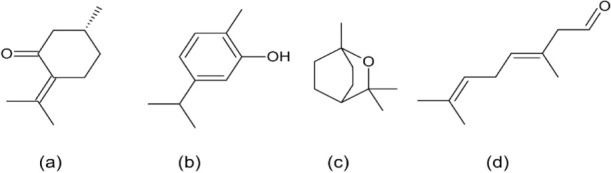
The most abundant compounds of *M. mollis* EO: **(a)** pulegone; **(b)** carvacrol; **(c)** 1,8-cineole; and **(d)** *(E)*-isocitral.

The EO was a transparent, light-amber liquid with a distinctive aromatic scent. The EO was analyzed by GC–MS, and 33 volatile compounds were detected, of which 30 were structurally identified and three unidentified. Together, all 33 peaks accounted for 100% of the total peak area, as determined by GC–FID normalization. (Table 1). The EO was dominated by oxygenated monoterpenes, with pulegone (63.82%) as the major component, followed by carvacrol (5.52%), 1,8-cineole (5.50%), and (*E*)-isocitral (4.98%) (Figure 3).

**TABLE 1:** Chemical composition of *Minthostachys mollis* EO as identified by GC–MS.

No	RT	LRI^a^	LRI^b^	Chemical compound	Chemical formula	%
1	8.764	936	932	α−Pinene	C_10_H_16_	0.63 ± 0.04
2	10.512	977	969	Sabinene	C_10_H_16_	1.09 ± 0.10
3	10.729	983	974	β-Pinene	C_10_H_16_	0.62 ± 0.08
4	11.175	989	979	1-Octen-3-ol	C_8_H_16_O	0.50 ± 0.02
5	13.005	1022	1020	ρ-Cymene	C_10_H_14_	1.23 ± 0.07
6	13.127	1025	1024	Limonene	C_10_H_16_	0.36 ± 0.04
7	13.348	1030	1026	1,8-Cineole	C_10_H_18_O	5.50 ± 0.21
8	13.947	1042	1044	(*E*)-β-Ocimene	C_10_H_16_	0.28 ± 0.02
9	16.773	1102	1095	Linalool	C_10_H_18_O	1.37 ± 0.15
10	16.981	1106	1110	1-Octen-3-yl acetate	C_10_H_18_O_2_	2.53 ± 0.17
11	19.800	1165	1159	Menthofuran	C_10_H_14_O	0.58 ± 0.07
12	19.991	1169	1158	Iso-Menthone	C_10_H_18_O	1.38 ± 0.11
13	20.613	1182	1177	(*E*)-Isocitral	C_10_H_16_O	4.98 ± 0.19
14	21.565	1202	1186	α-Terpineol	C_10_H_18_O	1.12 ± 0.11
15	22.562	1233	1234	3E-Decen-2-one	C_10_H_18_O	0.33 ± 0.02
16	23.698	1248	1233	Pulegone	C_10_H_16_O	63.82 ± 0.56
17	23.943	1243	1239	Carvone	C_10_H_14_O	0.53 ± 0.05
18	25.405	1285	1283	Isobornyl acetate	C_12_H_20_O_2_	0.69 ± 0.06
19	25.640	1299	1289	trans-Sabinyl acetate	C_12_H_20_O_2_	0.14 ± 0.01
20	26.459	1308	1298	Carvacrol	C_10_H_14_O	5.52 ± 0.24
21	26.810	1316	-	Unidentified	-	0.63 ± 0.05
22	28.323	1351	1340	Piperitenone	C_10_H_14_O	0.26 ± 0.02
23	29.279	1372	1366	Piperitenone oxide	C_10_H_14_O_2_	0.35 ± 0.03
24	29.592	1389	1387	β-Bourbonene	C_15_H_24_	0.29 ± 0.02
25	29.871	1396	-	Unidentified	-	0.03 ± 0.01
26	31.194	1417	1417	(*E*)-Caryophyllene	C_15_H_24_	1.54 ± 0.11
27	33.850	1480	1478	γ-Muurolene	C_15_H_24_	1.14 ± 0.10
28	34.462	1504	1500	Bicyclogermacrene	C_15_H_24_	0.82 ± 0.09
29	35.363	1517	1511	δ-Amorphene	C_15_H_24_	0.33 ± 0.07
30	35.687	1525	-	Unidentified	-	0.02 ± 0.01
31	37.907	1581	1574	Germacrene D-4-ol	C_15_H_26_O	0.35 ± 0.01
32	38.023	1584	1577	Spathulenol	C_15_H_24_O	0.79 ± 0.12
33	38.163	1588	1582	Caryophyllene oxide	C_15_H_24_O	0.26 ± 0.03
Monoterpene hydrocarbons (%)						4.21 ± 0.05
Oxygenated monoterpenes (%)						86.24 ± 0.24
Sesquiterpene hydrocarbons (%)						4.12 ± 0.10
Oxygenated sesquiterpenes (%)						1.40 ± 0.09
Other compounds (%)						4.04 ± 0.12
Total (%)						100.0

LRI^a^, linear retention index calculated; LRI^b^ linear retention index from references; % mean percentage content in the EO, over three determinations; SD, standard deviation; RT, retention time.

### 3.3 Enantioselective analysis

The enantiomeric distribution of all resolved chiral compounds is shown in Table 2. Two compounds, (1R,5R)-(+)-sabinene and (S)-(−)-limonene, were found to be enantiomerically pure. In contrast, α-pinene was present as a non-racemic mixture, with the (1*S*,5*S*)-(−)-enantiomer predominating (92.54%), corresponding to an enantiomeric excess (*e.e.*) of 84.97%. Similarly, β-pinene was resolved as a non-racemic mixture, with the (1*S*,5*S*)-(−)-enantiomer predominating at 80.63% (*e.e.* = 61.86%). Linalool was also present as a non-racemic mixture, with the (*R*)-(−)-enantiomer accounting for 76.57% of the total (*e.e.* = 53.50%).

**TABLE 2:** Enantiomeric distribution of the essential oil of *Minthostachys mollis* injected into 2,3-diacetyl-6-*tert*-butyldimethylsilyl-β-cyclodextrin**.**

Enantiomer	LRI	Distribution (%)	*e.e.* (%)
(1*S*,5S)-(−)-α-pinene	926	92.54	84.97
(1*R*,5*R*)-(+)-α-pinene	929	7.57	
(1*R*,5*R*)-(+)-sabinene	1008	100	100
(1*R*,5*R*)-(+)-β-pinene	977	18.77	61.86
(1S,5S)-(−)-β-pinene	979	80.63	
(*S*)-(−)-limonene	1058	100	100
(*R*)-(−)-linalool	1274	76.57	53.50
(*S*)-(+)-linalool	1280	23.06	

LRI, calculated linear retention index; Distribution (%) = relative proportion of each enantiomer with respect to the total amount; e. e. = enantiomeric excess, which provides a direct measure of chiral purity.

### 3.4 Acetylcholinesterase inhibition of M. mollis EO “muña”

**FIGURE 4:**
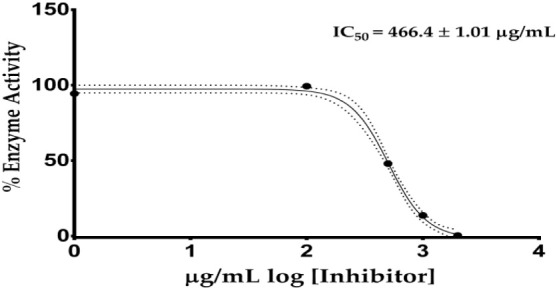
Acetylcholinesterase inhibitory activity of *M. mollis* EO. The IC_50_ values were calculated from concentration–response curves (mean ± SD, n = 3).

Figure 4 shows that the mean inhibitory concentration (IC_50_) of EO was 466.4 ± 1.01 μg/mL, which was calculated from three independent measurements. Donepezil was used as a positive control, with an IC_50_ value of 12.40 ± 0.03 μg/mL, under the same experimental conditions.

### 3.5 Acetylcholinesterase activity *in silico* of the volatile components of M. mollis EO

**FIGURE 5:**
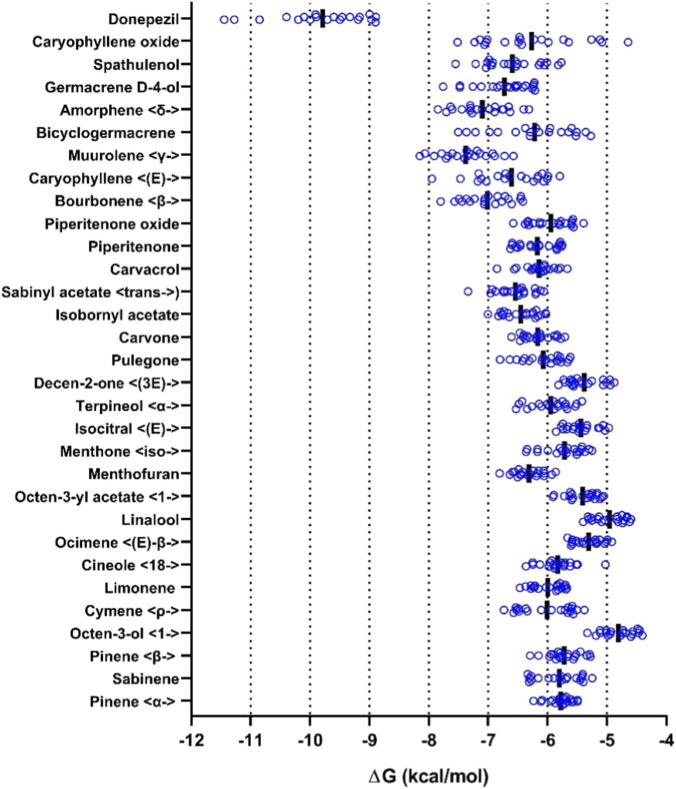
Molecular docking of the volatile constituents *M. mollis* EO with human AChE (PDB ID: 4EY7). Each blue circle in the figure represents an individual docking pose (n = 20 per ligand), and the black vertical line indicates the mean binding free energy expressed as ΔG, kcal/mol. Donepezil was used as a pharmacological reference inhibitor.

Molecular docking analysis using AutoDock Vina was performed to estimate the binding free energies (ΔG) of the 30 structurally identified volatile constituents of *M. mollis* EO on AChE (Figure 5). For each ligand, 20 docking poses were generated, and the distribution of binding energies and, mean ΔG values are shown. Donepezil (reference inhibitor) was included as a positive control due to its established clinical use as an AChE inhibitor. As expected, donepezil exhibited the highest binding affinity, with an average ΔG value of −9.7 kcal/mol and an RMSD of 0.84 Å for the best pose, relative to the crystallographic conformation. Among the *M. mollis* constituents, the sesquiterpenes muurolene, amorphene, and bourbonene exhibited the most favorable mean binding energies (−7.3, −7.09, and −7.0 kcal/mol, respectively), whereas 1-octen-3-ol displayed the weakest interaction (−4.8 kcal/mol). Overall, the docking results indicate that several sesquiterpene components of the EO interact with the AChE binding site with moderate affinity, although their predicted binding strengths are lower than donepezil.

**FIGURE 6:**
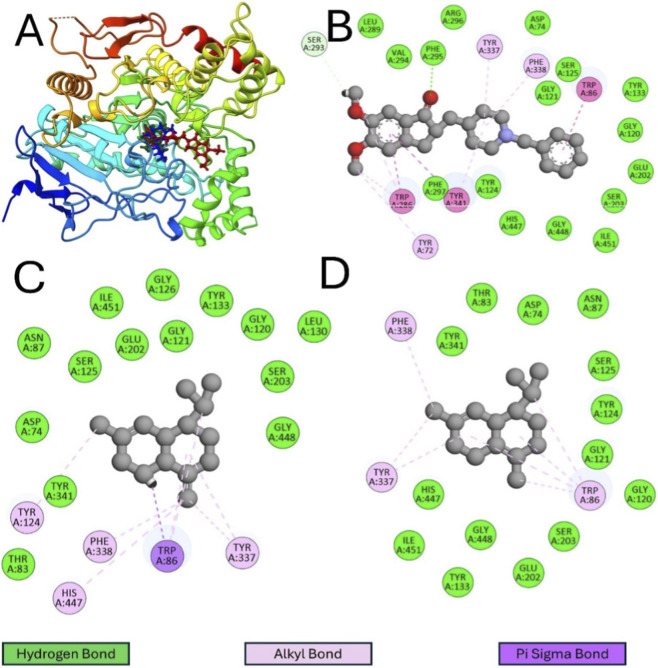
Analysis of molecular interactions at the binding site of the A chain of acetylcholinesterase. **(A)** Shows the binding of three molecules at the site: donepezil (red), γ-muurolene (green), and δ-amorphene (blue). **(B)** Highlights the interactions between donepezil and the enzyme, whereas **(C,D)** display the interactions with γ-muurolene and δ-amorphene, respectively.

Structural characterization of the protein–ligand complexes revealed unique interaction profiles between selected *M. mollis* constituents and human AChE (Figure 6). Three ligands were analyzed, donepezil (reference inhibitor), γ-muurolene, and δ-amorphene, which demonstrated better binding energies in the docking analysis. Figure 6A,B shows that donepezil exhibited a multifaceted interaction profile within the AChE binding gorge, forming several interactions, particularly a hydrogen bond with PHE295, along with several hydrophobic bonds with residues, including TRP286, TYR72, and TYR341. This interaction profile is partially similar to the binding mode described for donepezil, which interacts with both the catalytic active site and the peripheral anionic site of AChE. In contrast, γ-muurolene (Figure 6C) primarily interacted through hydrophobic interactions, forming eight nonpolar associations with aromatic and hydrophobic residues, including TYR124, PHE338, HIS447, TRP86, and TYR337. These results are consistent with the characteristics of hydrocarbon sesquiterpenes, no hydrogen bonds were observed in the analysis, and binding was mainly by van der Waals and π-alkyl interactions within the enzyme’s aromatic pocket. δ-amorphene (Figure 6D) exhibited a binding mechanism characterized by hydrophobic interactions, forming seven associations predominantly with residues such as TYR337, PHE338, and TRP86. The absence of hydrogen bonding interactions underscores the nonpolar nature of the ligand. In summary, while donepezil engages AChE through a combination of hydrogen bonding and hydrophobic interactions, γ-muurolene and δ-amorphene presented hydrophobic interactions to bind AChE. Notably, all three ligands interacted with residues located in both the catalytic region and the peripheral anionic site, suggesting a different molecular recognition profile than that of classical synthetic AChE inhibitors.

**FIGURE 7:**
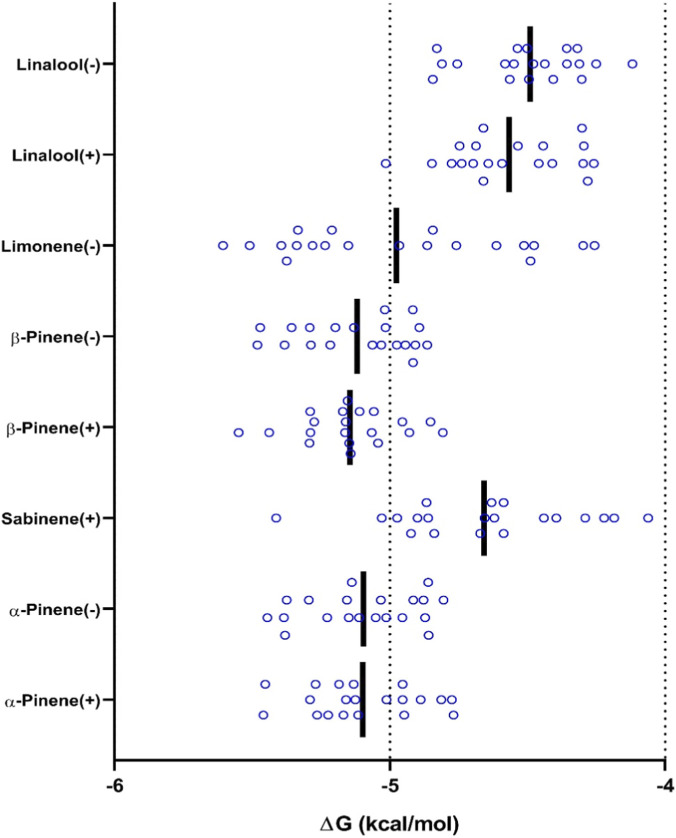
Molecular docking of chiral monoterpene enantiomers identified in *M. mollis* EO against AChE (PDB ID: 4EY7). Each blue circle represents an individual docking pose (n = 20), and the black vertical line indicates the mean binding-free energy (ΔG, kcal/mol). Dotted vertical lines at −5.0 and −4.0 kcal/mol are shown as references.

Enantioselective GC–MS analysis identified several chiral monoterpenes in *M. mollis* EO, prompting a comparative docking evaluation of their enantiomeric pairs. Molecular docking was performed for both enantiomers of α-pinene, β-pinene, and linalool, and for the single enantiomers exclusively detected for limonene [(*S*)-(−)] and sabinene [(1*R*,5*R*)-(+)] (Figure 7; Supplementary Figures S1, S2). The mean binding free energies of the enantiomeric pairs were virtually equivalent: α-pinene (−5.095 vs. −5.098 kcal/mol; ΔΔG = 0.003), β-pinene (−5.118 vs. −5.144 kcal/mol; ΔΔG = 0.026), and linalool (−4.490 vs. −4.567 kcal/mol; ΔΔG = 0.077). These differences are well within the uncertainty of the docking method, indicating that AChE binding affinity for these monoterpene scaffolds is not significantly modulated by the stereochemical configuration.

### 3.6 Molecular dynamics

**FIGURE 8:**
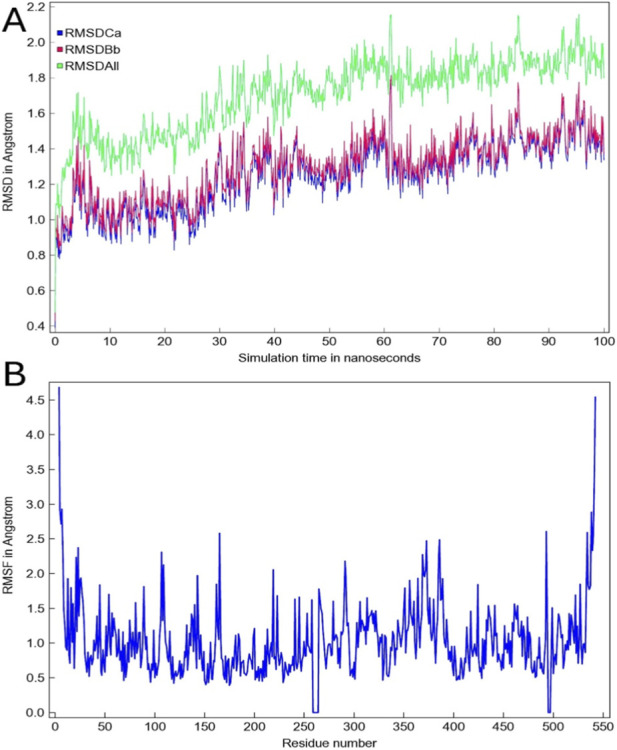
Molecular dynamics simulation of the γ-muurolene-AChE complex over 100 ns. **(A)** Root Mean Square Deviation (RMSD) of Cα atoms (blue), backbone atoms (red), and all heavy atoms (green) relative to the initial minimized structure, expressed in Ångströms (Å). **(B)** Per-residue Root Mean Square Fluctuation (RMSF) of AChE upon binding with γ-muurolene, highlighting regions of higher conformational flexibility, predominantly at the N- and C-termini.

The molecular dynamics of the complex formed between γ-muurolene and human AChE were examined over a 100 ns simulation in explicit water, and the results are showed in Figure 8. Panel A shows the root-mean-square deviation (RMSD) variations of the solute from its initial structure over time, calculated for the Cα atoms (blue), peptide backbone (red), and all heavy atoms (green). After a rapid increase during the first nanoseconds, associated with the relaxation of the minimized conformation, the trajectories of Cα and backbone stabilized within the range of approximately 1.0–1.6 Å, whereas the RMSD of all atoms fluctuated around 1.4–2.0 Å. This behavior indicates that the overall fold of AChE was preserved throughout the simulation and that the observed variations were mainly due to local conformational changes in the side chains and ligands in the active site. Panel B shows the per-residue root-mean-square fluctuation (RMSF) of the protein. This shows that the structural core is fairly rigid (with values between 0.5 and 1.7 Å) and that the N- and C-terminal ends and several exposed loops are very flexible, with values over 2.0 Å. The results of both simulations support the notion that the γ-muurolene–AChE complex remains structurally stable in solution. However, some flexible areas in the binding site environment might make it easier for the complex to change shape so that it can interact with other molecules in a favorable manner.

## 4 Discussion

The aerial parts of *M. mollis* collected in Ayacucho (Peru) were evaluated to a macroscopic analysis as part of the botanical characterization of this species. Thereafter, the EO obtained from the leaves was assessed for its organoleptic qualities, which are typically used as indicators of the quality and authenticity of the EO. The chemical composition of the EO was analyzed using GC–MS, which provided a detailed qualitative and quantitative profile of its volatile constituents.

In our study, pulegone was the most abundant volatile constituent with 63.82% ± 0.56% of the total EO, and minor sesquiterpenes such as γ-muurolene, δ-amorphene, and β-bourbonene were detected in relatively low proportions. A comparison with previously studies revealed considerable variability in the chemical compositions of *M. mollis* EOs from different geographical regions. For instance, the EO obtained from *M. mollis* collected in the Amazonas region of Peru was reported to contain nepetalactone (30.16%), linalool (9.63%), and D-limonene (6.87%) as the major constituents (Granda-Santos et al., 2025). In Argentina, studies on *M. mollis* EO have also revealed distinct chemical profiles, with menthone (51.2%), pulegone (31.8%), and isomenthone (3.1%) as the main constituents (Cravero Ponso et al., 2025). Similarly, Rojas-Molina et al. (2024) analyzed samples from Ecuador and identified carvacryl acetate (44.01%), carvacrol (16.51%), and menthone (8.20%) as the predominant components, whereas another Ecuadorian sample studied by Fernández et al. (2017) showed neomenthol (32.34%) as the main compound, followed by pulegone (28.42%) and menthone (19.32%). Additionally, Benites et al. (2018) described a different compositional profile for *M. mollis* leaves collected in Cajamarca, Peru, where menthone (13.2%), pulegone (12.4%), cis-dihydrocarvone (9.8%), and carvacrol acetate (8.8%) were identified as the most abundant constituents.

In contrast, the most relevant contribution of this study is the first enantioselective characterization of *M. mollis* EO. The exclusive presence of (*S*)-(−)-limonene and (1*R*,5R)-(+)-sabinene, along with the marked predominance of (1*S*,5*S*)-(−)-α-pinene and (1*S*,5*S*)-(−)-β-pinene, reveals a clear biosynthetic stereochemical preference. This is particularly relevant, because the biological activity of monoterpenes is often strongly dependent on their stereochemical configuration. Consequently, the enantiomeric composition of EO is an important parameter for their chemical characterization, potential biological effects, and quality control (Valarezo et al., 2025).

Regarding AChE inhibition, *M. mollis* EO exhibited moderate inhibitory activity *in vitro*, as showed by its IC_50_ value. The inhibitory activity of EO-based AChE inhibitors varies widely depending on their chemical composition and terpene distribution. For instance, the EO of *Pogostemon cablin*, characterized by a high sesquiterpene content, exhibited an IC_50_ of 24.15 *μ*g/mL (Jayaprakash et al., 2026). Similarly, EOs from *Citrus paradisi* (IC_50_ = 12.62 ± 0.48 μg/mL)*, Lawsonia inermis* (IC_50_ = 43.90 ± 0.97 *μ*g/mL) (Amawi and Ashmawy, 2026), *Croton alnifolius* (IC_50_ = 61.74 ± 1.02 *μ*g/mL) (Cruz et al., 2026), *Mentha spicata* (IC_50_ = 372.7 *μ*g/mL) (Chu et al., 2025), and *Eugenia valvata (*IC_50_ = 53.08 ± 1.13 *μ*g/mL) (Calva et al., 2023), have also been reported to exert AChE inhibitory effects with variable degrees of potency. Regarding pulegone monoterpenoid, the most abundant compound in the EO, previous studies have reported its individual AChE inhibitory activity, with an IC_50_ of 9.0 ± 0.41 mM. Compared to other terpenoids such as carvone (IC_50_ = 2.9 ± 0.12 mM) and ocimene (IC_50_ = 4.7 ± 0.20 mM), pulegone exhibited lower activity (Wojtunik-Kulesza et al., 2017), although it showed greater potency than 1,8-cineole (IC_50_ = 266.0 ± 11.87 mM). Nevertheless, EOs with high pulegone content, such as that of *Micromeria fruticosa* (pulegone 61.89%), have been reported to show an IC_50_ of 82.26 ± 0.80 μg/mL (Althaher and Oran, 2025), suggesting that the overall inhibitory activity of pulegone-rich EOs may not be solely attributable to this major constituent.

The inhibitory activity of the chemical constituents of *M. mollis* EO against human AChE was also explored using molecular docking, providing insights into their possible contribution to the observed *in vitro* activity. Among the 30 docked ligands, γ-muurolene, δ-amorphene, and β-bourbonene sesquiterpenes exhibited the most favorable binding free energies, although their predicted affinities were lower than that of the reference inhibitor donepezil (ΔG value of −9.7 kcal/mol), which is consistent with previously reported docking scores for donepezil against human AChE under comparable computational conditions, such as −8.60 ± 0.26 kcal/mol (Yang et al., 2025), and −8.8 kcal/mol (Laghchioua et al., 2024). Although the binding energy of γ-muurolene obtained in this study (−7.3 kcal/mol) was lower than that reported in another docking study (−9.134 kcal/mol) (Silva Júnior et al., 2024), its interaction profile observed within the aromatic gorge supports its potential contribution to enzyme inhibition. Previous studies have demonstrated that the biological activity of natural product mixtures may arise from interactions among constituents, in which even minor compounds can significantly modulate the overall effect (Bunse et al., 2022).

According to the structure–activity relationship, the hydrophobic sesquiterpene scaffold favors interactions within the aromatic gorge of AChE. The binding modes observed for γ-muurolene and δ-amorphene were mostly due to hydrophobic and π–alkyl interactions with aromatic residues such as TRP86, TYR337, PHE338, and TYR341, which are close to the enzyme’s catalytic gorge. In contrast, donepezil exhibited a more complex interaction pattern. It formed hydrogen bonds, especially with PHE295, and had many hydrophobic residues, as reported by Laghchioua et al. (2024), with interactions at TRP286, PHE297, TYR337, and TYR341. These differences show that synthetic inhibitors and nonpolar natural terpenes use different molecular recognition mechanisms. The frequent participation of residues such as TRP86 and TYR337, which are recognized for their significant roles in AChE inhibition, corroborates the contribution of these sesquiterpenes found in *M. mollis* EO to the overall inhibitory effect and corresponds with contemporary strategies focused on discovering multitarget or complementary inhibitors for neurodegenerative disorders such as AD (Makarian et al., 2021). Notably, this inverse relationship between relative abundance and predicted binding affinity, whereby minor sesquiterpene constituents outperformed the dominant component pulegone in docking scores, is consistent with observations in other EO systems. For instance, sesquiterpene alcohol-rich *Mentha aquatica* EO showed the most potent AChE inhibition among *Mentha* species, despite sesquiterpenes being present as minor constituents relative to the dominant menthol fraction (Miyazawa et al., 1998).

Furthermore, the proportional contribution of the identified active components to the overall AChE inhibitory activity of the EOs did not directly correlate with their relative abundance (Dohi et al., 2009). Together, these precedents support the interpretation that compositional predominance is not a reliable predictor of contribution to receptor-mediated enzyme inhibition, and that the sesquiterpene fraction of *M. mollis* EO may be considered a pharmacologically relevant contributor to the observed *in vitro* activity. Although some studies indicate that sesquiterpenes may possess favorable physicochemical characteristics owing to their lipophilicity, these predictions *in silico* require validation through cellular and *in vivo* studies. Safety and potential pharmacological interactions are crucial, particularly because approved AChE inhibitors are associated with dose-dependent peripheral side effects (Yang et al., 2024).

Regarding the chiral monoterpenes identified by enantioselective GC–MS, comparative docking analysis revealed no meaningful difference in AChE binding affinity between enantiomeric pairs (ΔΔG ≤0.134 kcal/mol), suggesting that stereochemical configuration does not significantly influence the interaction of these small non-polar monoterpene scaffolds with the AChE binding gorge.

Molecular dynamics simulations further supported the docking predictions by demonstrating that the γ-muurolene–AChE complex remained stable over a 100 ns trajectory. The backbone RMSD values stabilized below 2 Å after the initial equilibration phase, which is generally considered indicative of stable protein–ligand interactions (Hollingsworth and Dror, 2018). Moreover, the limited RMSF fluctuations observed in residues located within the catalytic gorge suggest that ligand binding does not induce significant conformational destabilization of the enzymes. Molecular dynamics simulations are widely used to validate docking-derived binding modes under dynamic and physiologically relevant conditions, as they account for solvent effects and conformational flexibility that are not captured by rigid docking approaches (Santos et al., 2019). Therefore, the molecular dynamic (MD) results reinforce the hypothesis that γ-muurolene can maintain a stable hydrophobic interaction within the human AChE binding site.

The development of natural AChE inhibitors as therapeutic candidates presents further translational challenges. Our study found that pulegone is the major constituent of the *M. mollis* EO. Therefore, its toxic effects must be carefully considered. In addition, pulegone has been linked to hepatotoxic effects at high exposure levels because metabolic bioactivation pathways generate reactive intermediates that can produce a liver damage (Nelson et al., 1992). Consequently, some regulatory agencies worldwide have established limits on the pulegone content in products for human consumption. For instance, the European Medicines Agency has suggested that herbal medicines containing pulegone should not be administered in amounts greater than 0.75 mg/kg body weight per day (Voigt et al., 2024). However, other aspects must be considered when analyzing the toxicological findings of pulegone-rich EOs from the *Minthostachys* species. These plants have a longstanding history of traditional therapeutic application in the Andean regions such as Peru, where it is typically consumed as diluted infusions for digestive disorders, with no reported evidence of toxicological effects. Recent studies have indicated that pulegone-rich EOs do not necessarily correlate directly with their cytotoxic or genotoxic effects, particularly when evaluated as a matrix. Escobar et al. (2012). showed that *Minthostachys verticillata* EO, which contains 60.5% pulegone, did not induce cytotoxicity in Vero cells or human peripheral blood mononuclear cells, and did not cause genetic damage in the bone marrow micronuclei of mice. These findings suggest that interactions among volatile constituents may modulate toxicity, and that the biological profile of the entire EO differs from that of its isolated major components, as supported by previous studies on *M. verticillata* EO (Escobar et al., 2012; 2015).

The mechanistic basis for this attenuation of toxicity may involve competitive substrate interactions at the level of hepatic cytochrome P450 enzymes responsible for pulegone bioactivation. Specifically, CYP2E1, CYP1A2, and CYP2C19 have been identified as the principal isoforms catalyzing the conversion of pulegone to menthofuran, with being CYP2E1 the most active isoform (Khojasteh-Bakht et al., 1999). Moreover, 1,8-cineole and carvacrol, which are constituents of *M. mollis* EO, are CYP substrates that compete for shared hepatic microsomal oxidative capacity; 1,8-cineole is a high-affinity substrate for CYP3A4 and CYP3A5 in human liver microsomes, with moderate inhibition of CYP3A4-mediated reactions. Carvacrol undergoes CYP1A2, CYP2A6, and CYP2B6-mediated metabolism in human liver microsomes (Zehetner et al., 2019). As noted by Zehetner et al. (2019), when an EO is used as a whole mixture, all its constituents, including those present in low quantities, participate simultaneously in CYP interactions, and the contribution of each individual component to the final metabolic outcome remains difficult to predict from isolated-compound studies alone. This principle supports the hypothesis that the metabolic landscape of the entire *M. mollis* EO matrix may modulate the rate of CYP-mediated bioactivation of pulegone, potentially attenuating the generation of hepatotoxic reactive intermediates relative to that generated from pulegone in isolation.

The present study had several limitations, the *M. mollis* EO was only tested *in silico* and *in vitro*, the absence of *in vivo* studies limits its direct extrapolation to clinical application. Therefore, future *in vivo* studies in animal models are necessary to observe these pharmacological effects. In the docking analysis, the compounds were evaluated using a rigid receptor and did not evidence the complex interactions that EOs could achieve as a complex mixture of volatile compounds, which might synergize or antagonize the effect on AChE, particularly between pulegone and the minor sesquiterpene components. Regarding enantioselective analysis, it was limited to five chiral compounds due to the restricted availability of enantiomerically pure standards, and additional chiral constituents may remain uncharacterized. In addition, it should be noted that the triplicate measurements in the AChE inhibition assay performed in this study represent technical replicates obtained from a single EO batch, and biological replicates from independently collected plant material were not included.

## 5 Conclusion

The present study provides a comprehensive chemical and enantioselective characterization of *M. mollis* EO from Peru. GC–MS analysis revealed a chemical profile dominated by oxygenated monoterpenes, with pulegone as the major volatile constituent. Enantioselective GC–MS demonstrated a clear stereochemical preference for specific monoterpene enantiomers, highlighting the non-racemic nature of key components, such as α-pinene, β-pinene, and linalool. These findings provide novel chemical information relevant to the quality assessment and standardization of *M. mollis* EO. *In vitro* assays showed that EO exerted a moderate inhibitory effect on AChE, which is consistent with the molecular docking results, indicating that sesquiterpene constituents interact with the enzyme mainly through hydrophobic bonds within the catalytic and peripheral anionic sites. Although the observed inhibitory activity was lower than that of donepezil, combined *in vitro* and *in silico* evidence suggests that sesquiterpenes contribute to the AChE inhibitory activity of EO. Further studies *in vivo* are needed to establish the pharmacological relevance of these findings. However, considering that pulegone is the major constituent and is associated with dose-dependent toxicity, these findings should be interpreted with caution and not as evidence of direct therapeutic applications.

## Data Availability

The original contributions presented in the study are included in the article/Supplementary Material, further inquiries can be directed to the corresponding author.
