# Supplementary material for: Chemical and enantioselective GC–MS characterization of Minthostachys mollis (Benth.) Griseb. Essential oil from Peru and its acetylcholinesterase inhibitory activity
Source: Front Pharmacol. 2026 Jun 24;17:1864128. doi: 10.3389/fphar.2026.1864128 (PMC13341438; doi:10.3389/fphar.2026.1864128)
Supplement: Supplementary file 1 [file DataSheet1.PDF]

Supplementary Material

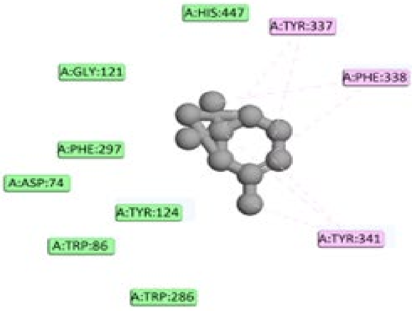

α-Pinene(+)

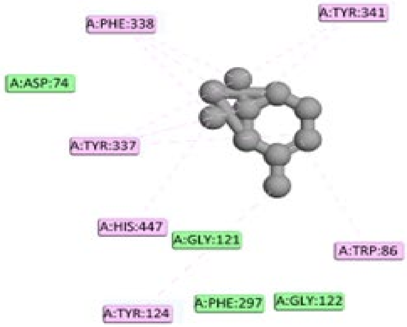

α-Pinene(-)

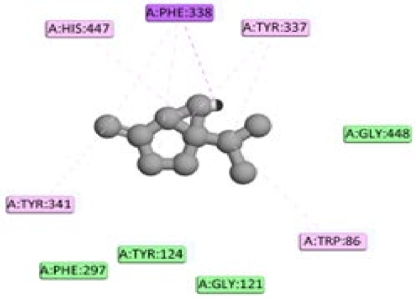

Sabinene(+)

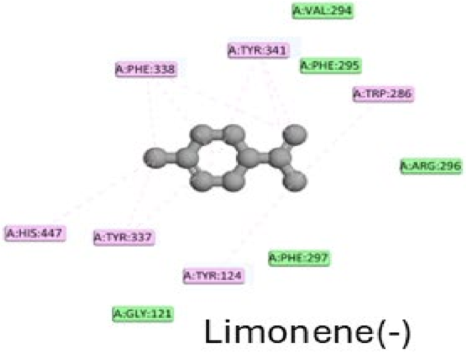

Limonene(-)

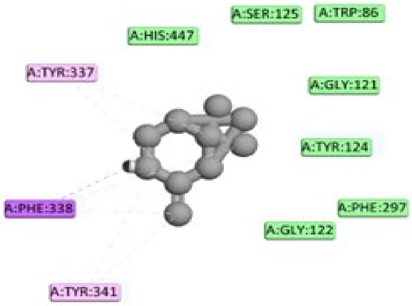

β-Pinene(+)

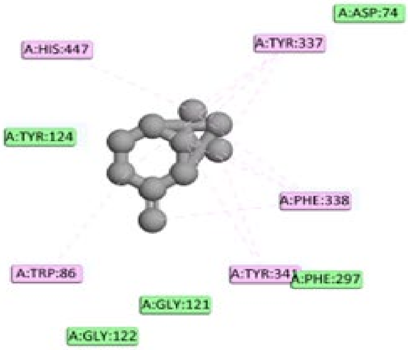

β-Pinene(-)

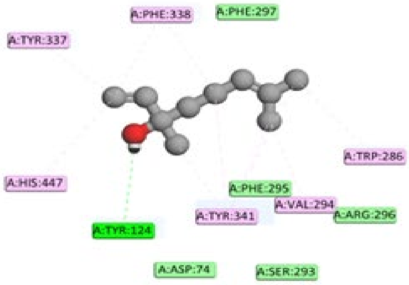

Linalool(+)

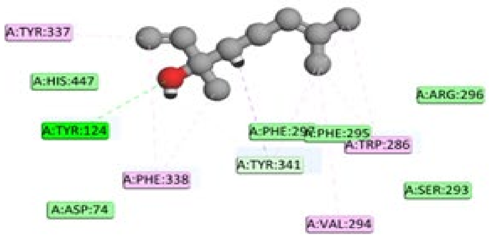

Linalool(-)

Supplementary Figure S1. Two-dimensional protein–ligand interaction profiles of chiral monoterpene enantiomers identified in *M. mollis* EO docked into human AChE (PDB ID: 4EY7).

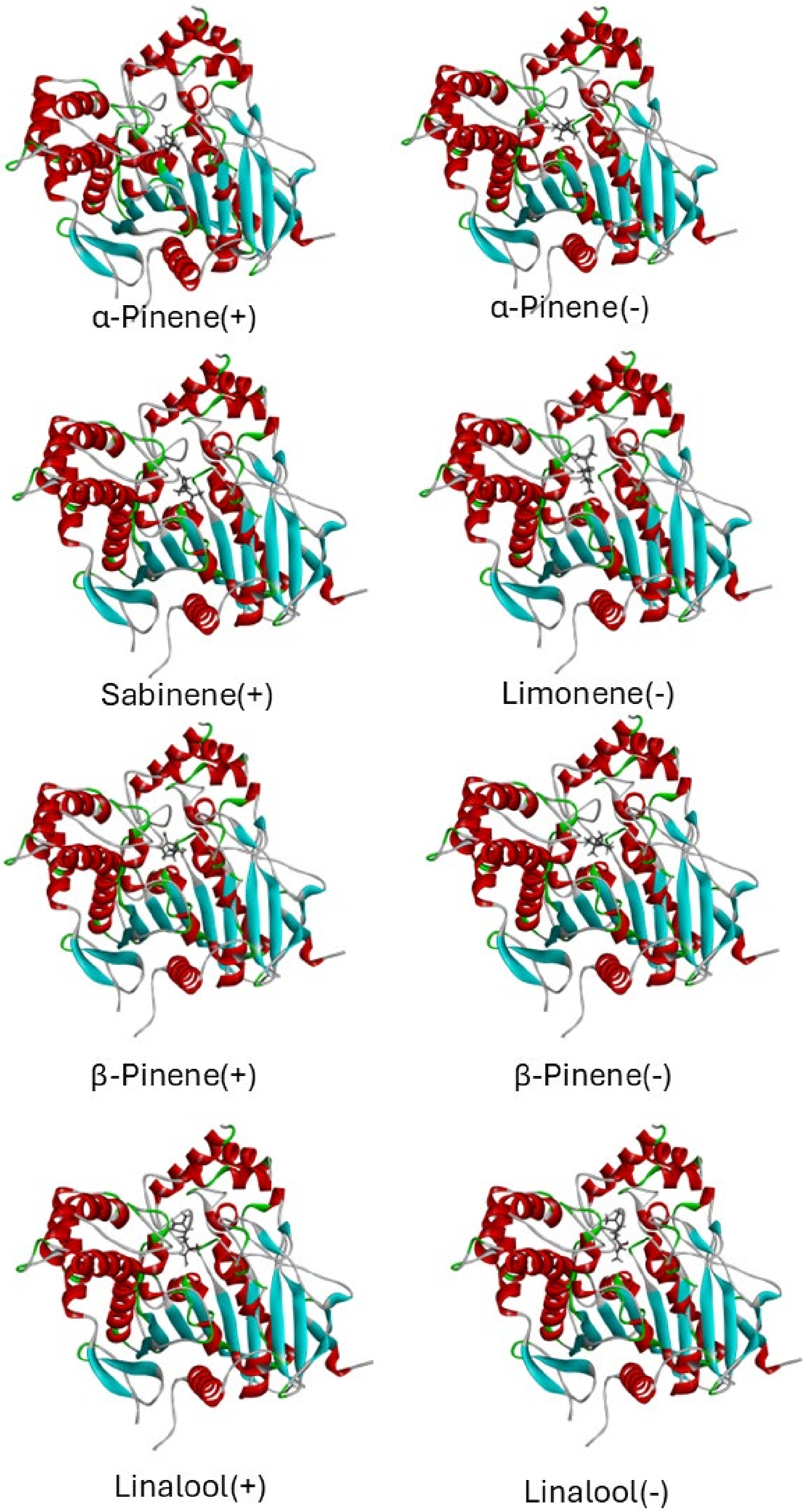

Supplementary Figure S2. Three-dimensional binding poses of chiral monoterpene enantiomers within the AChE binding gorge (PDB ID: 4EY7). The protein structure is represented as ribbons (red α-helices and cyan β-sheets); ligands are shown as stick models.
